# A Laboratory Handbook of Physiologic Chemistry and Urine-Examination

**Published:** 1902-02

**Authors:** 


A LABORATORY HANDBOOK OF PHYSIOLOGIC CHEMISTRY AND URINE-EXAMINATION. By CHARLES G. L. WOLF, M. D., Instructor in Physiologic Chemistry, Cornell University Medical College, New York. Duodecimo volume of 190 pages, fully illustrated. Philadelphia and London: W. B. Saunders & Co. 1901. [Price, cloth, $1.25 net.]

A book for students containing in the first part laboratory exercises in physiological chemistry, which afford a broad basis for the practical clinical tests in the latter half of the volume.

The tests for examination of the gastric contents and the urine have been selected with care, none being given which are not suitable for clinical work. A brief space in the text is devoted to urinary and gastric diagnosis, and the volume is concluded with carefully compiled tables for urinary diagnosis. Altogether the book, while hardly meeting the needs of the practitioner, will prove of great service to the student of medicine.—M.J.F.

